# Phase Evolution in the CaZrTi_2_O_7_–Dy_2_Ti_2_O_7_ System: A Potential Host Phase for Minor Actinide Immobilization

**DOI:** 10.1021/acs.inorgchem.1c03816

**Published:** 2022-04-04

**Authors:** Lewis R. Blackburn, Luke T. Townsend, Sebastian M. Lawson, Amber R. Mason, Martin C. Stennett, Shi-Kuan Sun, Laura J. Gardner, Ewan R. Maddrell, Claire L. Corkhill, Neil C. Hyatt

**Affiliations:** † Department of Materials Science and Engineering, Immobilisation Science Laboratory, University of Sheffield, Sir Robert Hadfield Building, Mappin Street, Sheffield S1 3JD, U.K.; ‡ GeoRoc International (GRI) Ltd, Whitehaven, Cumbria CA28 8PF, U.K.; § School of Materials Science and Energy Engineering, Foshan University, Foshan 528000, China; ∥ National Nuclear Laboratory, Workington, Cumbria CA20 1PJ, U.K.

## Abstract

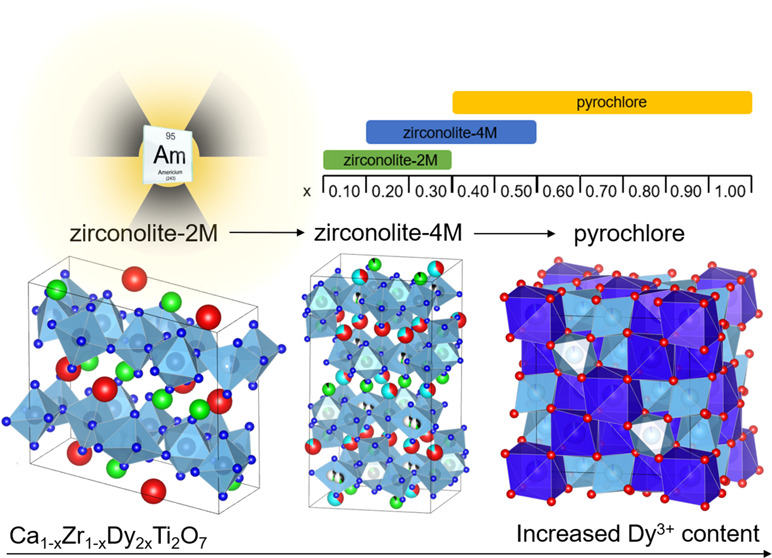

Zirconolite is considered to be a suitable wasteform material for the immobilization of Pu and other minor actinide species produced through advanced nuclear separations. Here, we present a comprehensive investigation of Dy^3+^ incorporation within the self-charge balancing zirconolite Ca_1–*x*_Zr_1–*x*_Dy_2*x*_Ti_2_O_7_ solid solution, with the view to simulate trivalent minor actinide immobilization. Compositions in the substitution range 0.10 ≤ *x* ≤ 1.00 (Δ*x* = 0.10) were fabricated by a conventional mixed oxide synthesis, with a two-step sintering regime at 1400 °C in air for 48 h. Three distinct coexisting phase fields were identified, with single-phase zirconolite-2M identified only for *x* = 0.10. A structural transformation from zirconolite-2M to zirconolite-4M occurred in the range 0.20 ≤ *x* ≤ 0.30, while a mixed-phase assemblage of zirconolite-4M and cubic pyrochlore was evident at Dy concentrations 0.40 ≤ *x* ≤ 0.50. Compositions for which *x* ≥ 0.60 were consistent with single-phase pyrochlore. The formation of zirconolite-4M and pyrochlore polytype phases, with increasing Dy content, was confirmed by high-resolution transmission electron microscopy, coupled with selected area electron diffraction. Analysis of the Dy L_3_-edge XANES region confirmed that Dy was present uniformly as Dy^3+^, remaining analogous to Am^3+^. Fitting of the EXAFS region was consistent with Dy^3+^ cations distributed across both Ca^2+^ and Zr^4+^ sites in both zirconolite-2M and 4M, in agreement with the targeted self-compensating substitution scheme, whereas Dy^3+^ was 8-fold coordinated in the pyrochlore structure. The observed phase fields were contextualized within the existing literature, demonstrating that phase transitions in CaZrTi_2_O_7_–REE^3+^Ti_2_O_7_ binary solid solutions are fundamentally controlled by the ratio of ionic radius of REE^3+^ cations.

## Abstract 2

Zirconolite (CaZrTi_2_O_7_) ceramics are candidate wasteform materials for Pu and other minor actinides. Herein, the Ca_1−*x*_Zr_1−*x*_Dy_2*x*_Ti_2_O_7_ solid solution was fabricated by a conventional mixed oxide synthesis, with Dy^3+^ included as a structural simulant for Am^3+^. A phase transformation from zirconolite-2M to zirconolite-4M was observed at low Dy concentrations (0.20 ≤ *x* ≤ 0.30) after which cubic pyrochlore was stabilized as the dominant phase. Observations and interpretations were supported by electron diffraction and X-ray absorption spectroscopic methods.

## 1 Introduction

Zirconolite structured materials have been widely studied for the immobilization of actinide-rich radioactive waste streams, due to excellent chemical alteration resistance and radiation tolerance.^1−5^ This includes use as a major constituent of the various SYNROC assemblages for the disposition of high-level actinide-rich wastes derived from nuclear fuel reprocessing.^6,7^ The CaZrTi_2_O_7_ parent structure has been shown to accommodate U, Pu, Np, and Cm and is therefore a suitable host matrix for minor actinide (MA) species such as Am.^8−11^ Natural analogue specimens have also been shown to retain ∼20 wt % U/Th over geological timescales.^12^ The parent structure ABC_2_O_7_ is derivative of the anion deficient fluorite structure type and is closely related to the pyrochlore A_2_B_2_O_7_ family of minerals. The ideal zirconolite unit cell is composed of planes of corner-sharing CaO_8_ and ZrO_7_ polyhedra, interleaved by hexagonal tungsten bronze (HTB)-style motifs along the [001] direction.^13^ Ti^4+^ cations are distributed across three distinct sites in the HTB plane: two Ti^4+^ sites are arranged in TiO_6_ octahedra and one Ti^4+^ is 5-fold coordinated in a 50% statistically coordinated trigonal bipyramidal site. In this idealized structure description, cation and HTB layers are integrated 1:1 along [001], related by a 180° rotation along the c* axis. Due to the two-layered structure, stoichiometric CaZrTi_2_O_7_ is referred to as the zirconolite-2M polytype, with reference to the monoclinic symmetry of the unit cell; this polytype has previously been demonstrated to crystallize over the compositional range CaZr*_x_*Ti_3–*x*_O_7_ for 0.8 < *x* < 1.3.^14^

As the zirconolite structure has three distinct cation acceptor sites, the solubility of REE^3+^/Ac^4+^ species is extensive. The incorporation of Ce^4+^/U^4+^/Pu^4+^ species within the Zr^4+^ site in zirconolite is accommodated by several structural transitions, first from zirconolite-2M to the zirconolite-4M polytype.^8,9,15^ The zirconolite-4M polytype is described by Coelho et al. as an admixture of zirconolite-2M and pyrochlore; four HTB-type layers interlaced with Ca/Zr polyhedra (zirconolite), and Ca/Ti polyhedra (pyrochlore), resulting in unit cell doubling along the *c*-axis from ∼11 to 23 Å.^16^ Further isovalent substitution of cations within the Zr^4+^ site promotes a structural transformation from zirconolite-4M to pyrochlore, although it should be noted that this does not occur for the corresponding CaZr_1–*x*_Th*_x_*Ti_2_O_7_ solid solution, for which the intermediate 4M phase does not form.^17^ Cubic pyrochlore-structured materials (parent structure A_2_B_2_O_7_—space group *Fd*3̅*m*; *Z* = 8) have attracted significant interest in many areas of solid-state chemistry, with titanate and zirconate pyrochlores (A_2_Ti_2_O_7_ and A_2_Zr_2_O_7_, respectively) developed as potential wasteforms for actinides, due to high radiation stability.^18−24^ The rare earth pyrochlore structure is derived from the fluorite (AO_2_) superstructure, with one-eighth of the oxygen atoms replaced by vacancies, and the A^3+^ and B^4+^ cations in 8- and 6-fold coordination with oxygen, respectively. These cations are ordered along the [110] direction, resulting in the unit cell adopting cubic symmetry. The phase stability of A_2_B_2_O_7_-type structures is dependent on the ionic radius ratio of the A and B cations; the ordered cubic pyrochlore structure is stable in the range 1.46 < *r*_A_/*r*_B_ < 1.78. Compounds with *r*_A_/*r*_B_ below this range adopt a disordered defect-fluorite structure, while compounds with *r*_A_/*r*_B_ > 1.78 crystallize with monoclinic layered perovskite-related structure.^19^

The present study aims to systematically evaluate the phase transitions in the CaZrTi_2_O_7_–Dy_2_Ti_2_O_7_ system with the progressive accommodation of Dy^3+^, acting as a surrogate for actinide species such as Pu^3+^, Am^3+^, Cm^3+^, and Np^3+^. These data are expected to complement existing data for closely related zirconolite solid solutions Ca_1–*x*_Zr_1–*x*_Gd_2*x*_Ti_2_O_7_, Ca_1–*x*_Zr_1–*x*_Y_2*x*_Ti_2_O_7_, Ca_1–*x*_Zr_1–*x*_Nd_2*x*_Ti_2_O_7_, Ca_1–*x*_Zr_1–*x*_Sm_2*x*_Ti_2_O_7_, and Ca_1–*x*_Zr_1–*x*_Ce_2*x*_Ti_2_O_7+δ_.^4,25−28^ While the incorporation of Dy^3+^ within the zirconolite structure has not been previously reported, the Dy_2_Ti_2_O_7_ pyrochlore end member has attracted notable interest given its applications as a spin-ice compound, due to prominent geometric frustration.^29,30^ Furthermore, Dy has been previously used as a surrogate for Am for the fabrication of AmN and (Am-Pu)N compounds, on the basis of ionic radii constraints and expediency (Am^3+^ = 1.09 Å; Dy^3+^ = 1.03 Å in 8-fold coordination).^31,32^

## 2 Experimental Procedure

### 2.1 Materials Synthesis

All materials used were fabricated by a conventional solid-state synthesis route from component oxides, targeting the solid solution Ca_1–*x*_Zr_1–*x*_Dy_2*x*_Ti_2_O_7_ (0.10 ≤ *x* ≤ 1.00, Δ*x* = 0.10). Precursors CaTiO_3_ (Sigma-Aldrich, 99.9%), ZrO_2_ (Sigma-Aldrich, 99.9%), TiO_2_ (anatase—Sigma-Aldrich, 99.9%) dried at 180 °C, and Dy_2_O_3_ (Alfa Aesar, 99.9%) dried at 800 °C were weighed according to the targeted composition, to yield 3 g batches. The oxide reagents were added to a ZrO_2_-lined milling jar and homogenized with Y-stabilized ZrO_2_ milling media and isopropanol for 20 min, using a Fritsch Pulverisette-23, operating at 25 Hz for 20 min. For each composition, the powder slurries were discharged, sieved to separate milling media, and dried at 80 °C overnight to evaporate excess solvent. Approximately 0.5 g of each composition was prepared for sintering by first compacting into the walls of a hardened steel die, under 3 tonnes of uniaxial pressure, forming powder compacts 13 mm in diameter. The pellets were then placed onto a zirconia crucible and sintered in air at 1400 °C (Δ5 °C min^–1^) for 24 h. Once cooled, the pellets were reground using a pestle and mortar, repressed into pellets, and subjected to a second sintering regime for a further 24 h at 1400 °C (Δ5 °C min^–1^) to promote phase purity. After the second sintering step, the pellets were recovered from the furnace for analysis.

### 2.2 Materials Characterization

Powder X-ray diffraction (XRD) was conducted using a Bruker D2 Phaser fitted with a Lynxeye position-sensitive detector. Data were acquired in the range 5° ≤ 2θ ≤ 80° (Δ0.02°) using Cu Kα radiation (λ = 1.5418 Å, Ni Filter), operating at 30 kV and 10 mA. Phase identification was achieved using the PDF4+ database. Quantitative phase analysis and unit cell dimensions were calculated by Rietveld analysis of powder XRD data, using the Bruker TOPAS software package. Prior to analysis by scanning electron microscopy (SEM), sintered pellets were mounted in cold setting resin and polished to a 1 μm optical finish. SEM data were collected using a Hitachi TM3030 operating with a 15 kV accelerating voltage at a working distance of 8 mm. Energy-dispersive X-ray spectrometry (EDS) for semiquantitative compositional analysis was conducted using a Bruker Quantax 70 spectrometer. EDS mapping was performed over an area of 140 × 105 μm^2^ for approximately 10 min.

X-ray absorption spectroscopy (XAS) at the Dy L_3_-edge was conducted at the Photon Factory Synchrotron Facility (Tsukuba, Japan) using beamline BL-27B, in a conventional transmission configuration. Spectra were collected at the Dy L_3_-edge (7790 eV) for specimens corresponding to nominal composition *x* = 0.10, 0.30, 0.60, and 1.00. Spectra were collected between 7590 and 8540 eV at the following steps, with a count time of 1s/step: 5 eV (7590–7760 eV), 0.5 eV (7760–7860 eV), 1 eV (7860–7940 eV), and 2 eV (7940–8540 eV). Samples were finely ground and dispersed in poly(ethylene glycol), to a concentration corresponding to one absorption length, and pressed into 13 mm pellets. Spectra were collected alongside Dy_2_O_3_, Dy_2_TiO_5_, and Dy_2_Ti_2_O_7_ reference compounds, representing Dy^3+^ in 6-fold, 7-fold, and 8-fold coordination with oxygen, respectively. Calibration of all XAS data was performed by aligning the Dy_2_O_3_ reference compound from this dataset to a previously collected Dy_2_O_3_ standard that was calibrated using a Co foil (edge position 7709 eV). All edge positions were chosen as the peak of the first derivative. Data reduction and fitting of the Dy L_3_ edge EXAFS were performed using Artemis and Athena with FEFF6.^33^ Best-fit models were informed by pyrochlore and zirconolite-2M structures from Farmer et al.^34^ and Whittle et al.,^35^ respectively. The F-test methodology for EXAFS was employed to test whether the addition of various shells was valid and statistically improved the fit, the results of which are denoted by α.^36^ For α, >67% is equal to 1σ and >95% is equal to 2σ in terms of standard deviation.

Raman spectra were collected for each sample using a Horiba-XploRA Plus system, operating with a 532 nm air-cooled Ar^+^ laser line, with a laser power of 5 mW. Powdered specimens were flattened onto a glass slide and placed incident to the laser line, with spectra collected in the range 100–1100 cm^–1^. Transmission electron microscopy (TEM) was undertaken using either a JEOL F200 or an FEI Tecnai T20, both operating at 200 keV, with both micrographs and electron diffraction (ED) patterns taken using a CCD camera. Specimens were prepared for TEM *via* the crushed grain powdered route, whereby a small amount of powder was rapidly ground in isopropanol and the resultant solution pipetted onto a holey lined Cu mesh grid.

## 3 Results and Discussion

### 3.1 Systematic Examination of Phase Evolution in the Ca_1–*x*_Zr_1–*x*_Dy_2*x*_Ti_2_O_7_ (0.10 ≤ *x* ≤ 1.00) System

**Figure 1:**
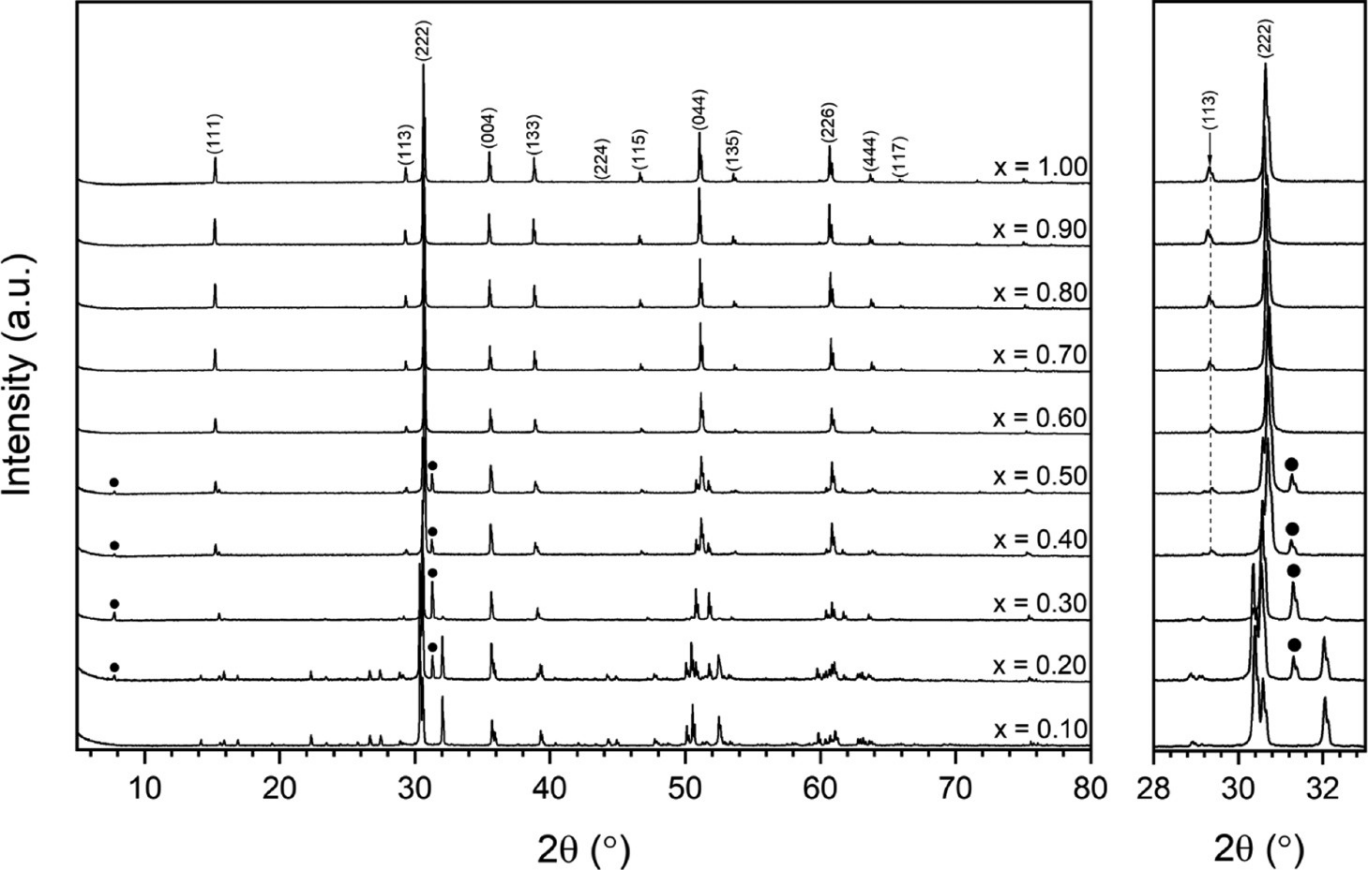
Powder X-ray diffraction data for Ca_1–*x*_Zr_1–*x*_Dy_2*x*_Ti_2_O_7_ specimens in the compositional range (0.0 ≤ *x* ≤ 1.0). The (002) and (008) zirconolite-4M reflections are highlighted by closed circles (●). Pyrochlore reflections are indexed by relevant (*hkl*) indices.

**Figure 2:**
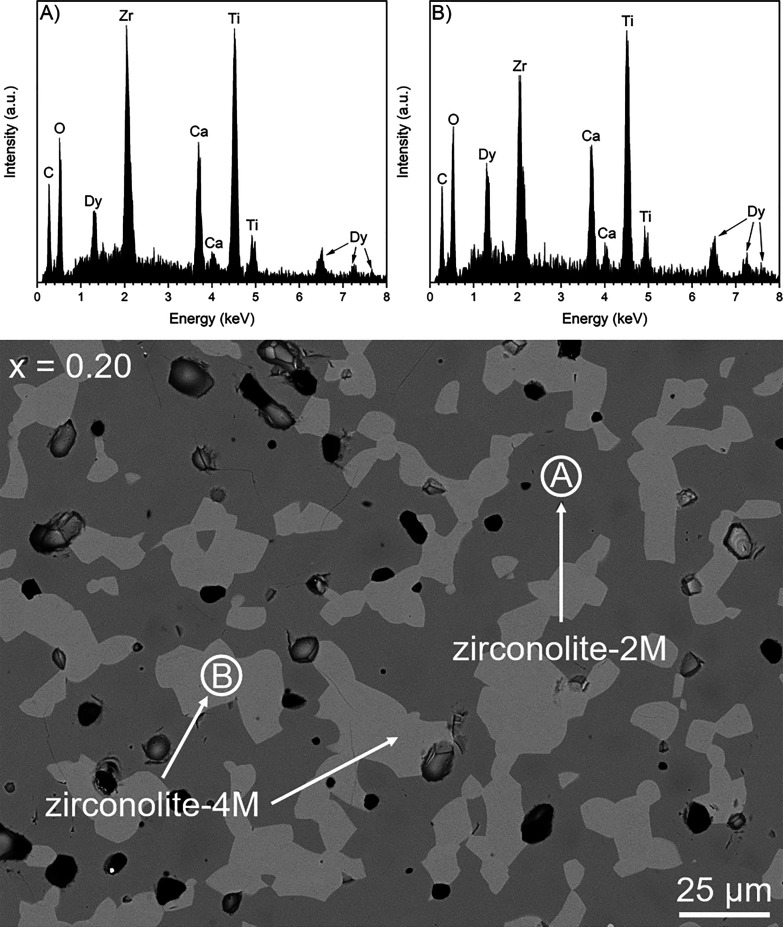
Backscattered electron micrograph of the *x* = 0.20 composition, with EDS spectra of zirconolite-2M and zirconolite-4M polytype phases.

**Figure 3:**
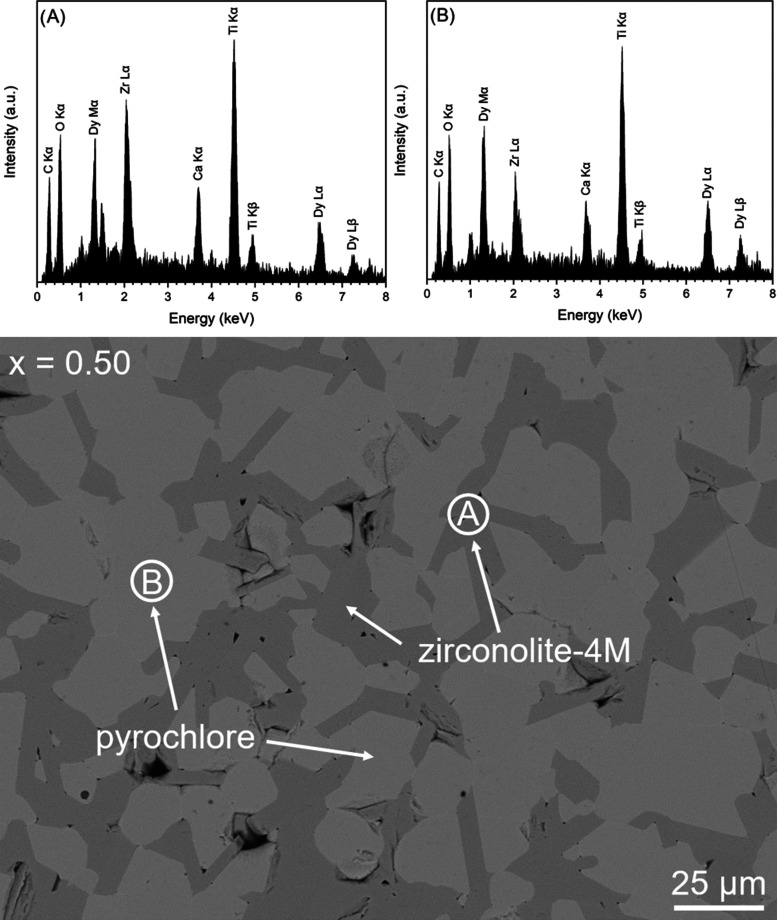
Backscattered electron micrograph of the *x* = 0.50 composition, with EDS spectra of zirconolite-4M and cubic pyrochlore phases.

**Figure 4:**
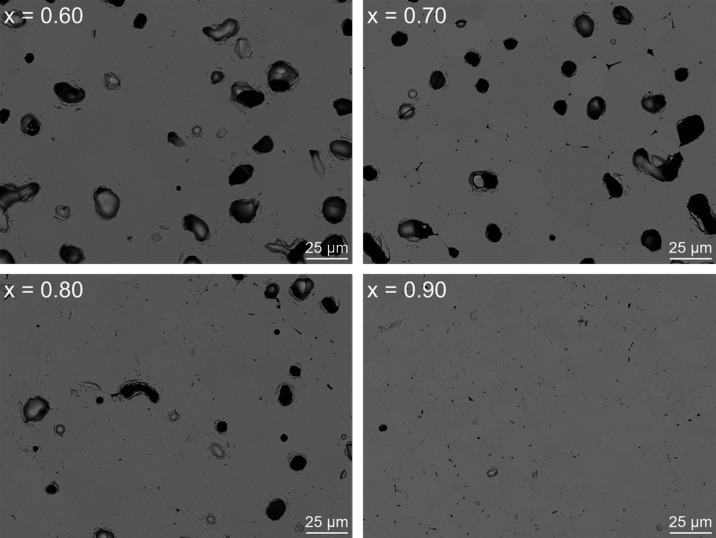
Backscattered electron micrographs of the *x* = 0.60, 0.70, 0.80, and 0.90 compositions, demonstrating phase-pure pyrochlore.

The phase evolution of Ca_1–*x*_Zr_1–*x*_Dy_2*x*_Ti_2_O_7_ ceramics was analyzed by powder X-ray diffraction (Figure 1). Three distinct phase fields were identified, corresponding to mixtures of zirconolite-2M (*C*2/*c*), zirconolite-4M (*C*2/*c*), and cubic pyrochlore (*Fd*3̅*m*). The *x* = 0.10 composition was found to form single-phase zirconolite-2M, characterized by the doublet at 2θ = 30.5° corresponding to the (221) and (40-2) reflections, the (004) reflection at 2θ = 31.9°. Unit cell dimensions (Table 1) as determined by Rietveld analysis were in agreement with those reported for closely related Ca_1–*x*_Zr_1–*x*_Gd_2*x*_Ti_2_O_7_ solid solutions.^25^ A structural transformation from zirconolite-2M to the zirconolite-4M polytype was observed in the compositional range 0.20 ≤ *x* ≤ 0.30, as characterized by the appearance of intense supercell reflections at 2θ = 7.8 and 31.1°, attributed to the (002) and (008) reflections in the zirconolite-4M structure, respectively. A representative section of the microstructure for the sample corresponding to *x* = 0.20 is given in Figure 2. Two distinct phases were distinguished by variation in backscattered electron contrast, identified to be zirconolite-2M and zirconolite-4M, in agreement with powder XRD data. The phase labeled **A** was determined by EDS analysis to be zirconolite-2M. The phase labeled **B** was consistent with zirconolite-4M, appearing brighter than the bulk matrix, given the expected higher solubility of Dy^3+^ in the 4M polytype. The average composition of both zirconolite phases was derived from semiquantitative EDS analysis and is in general agreement with targeted nominal stoichiometry (Table 2). The zirconolite-2M phase accounted for just 19.0 ± 0.8 wt % of the overall phase assemblage when targeting *x* = 0.30, yet, a two-phase mixture of zirconolite-4M and cubic pyrochlore was observed in the range 0.40 ≤ *x* ≤ 0.50; hence, zirconolite-4M was not isolated as a single phase in the solid solution. This was unsurprising, as the 4M phase has only previously been reported to crystallize in single phase over a narrow compositional range, sensitive to preparation route, ionic radii of dopant/host site, and targeted solid solution regime of REE^3+^/Ac^4+^.^8,9,16^ The cubic pyrochlore phase was evidenced by the appearance of reflections at 2θ = 15.1 and 29.2°, corresponding to reflections in the (111) and (113) plane, appearing at *x* = 0.40. The microstructure of the *x* = 0.50 sample is shown in Figure 3 and clearly displays a microstructure dominated by two phases. The phases were confirmed by EDS analysis to be zirconolite-4M and cubic pyrochlore (labeled as **A** and **B** in Figure 3, respectively) Additional reflections were not observed for 0.60 ≤ *x* ≤ 1.00, indicating complete Dy_2_O_3_ substitution within the zirconolite-pyrochlore mixture, and confirmed by SEM analyses (Figure 4).

**Table 1:** Quantitative Phase Analysis and Unit Cell Parameters of Each Phase as Determined from Rietveld Analysis of Powder X-ray Diffraction Data

		unit cell parameters				
nominal composition	phase assemblage (wt %)	*a* (Å)	*b* (Å)	*c* (Å)	β (deg)	*V* (Å^3^)
*x* = 0.10	zirconolite-2M^a^	12.47925(30)	7.28070(17)	11.37370(28)	100.6002(13)	1015.752(42)
*x* = 0.20	zirconolite-2M (64.9 ± 0.2)	12.49392(54) - 2M	7.28442(32) - 2M	11.37380(51 ) - 2M	100.6038(23) - 2M	1017.463(77) - 2M
	zirconolite-4M (35.1 ± 0.2)	12.50991(76) - 4M	7.17395(43) - 4M	22.95916(81 ) - 4M	84.8136(48) - 4M	2052.04(19) - 4M
*x* = 0.30	zirconolite-2M (19.0 ± 0.8)	12.5102(23) - 2M	7.2721(13) - 2M	11.3692(20) - 2M	100.465(23) - 2M	1017.10(32) - 2M
	zirconolite-4M (81.0 ± 0.8)	12.45916(35) - 4M	7.19043(26) - 4M	22.97562(45) - 4M	84.8373(26) - 4M	2049.96(10) - 4M
*x* = 0.40	zirconolite-4M (47.8 ± 0.7)	12.4532(31) - 4M	7.1922(18) - 4M	23.0213(27) - 4M	84.790(17) - 4M	2053.39(77) - 4M
	pyrochlore (52.2 ± 0.7)	10.10079(87) - P				1030.54(27) - P
*x* = 0.50	zirconolite-4M (44.7 ± 0.6)	12.4531(22) - 4M	7.19229(90) - 4M	23.0140(21) - 4M	84.861(11) - 4M	2052.99(48) - 4M
	Pyrochlore (55.3 ± 0.6)	10.10033(86) - P				1030.40(26) - P
*x* = 0.60	pyrochlore^a^	10.10258(37)				1031.09(11)
*x* = 0.70	pyrochlore^a^	10.10736(15)				1032.555(47)
*x* = 0.80	pyrochlore^a^	10.11497(15)				1034.890(47)
*x* = 0.90	pyrochlore^a^	10.12357(13)				1037.530(40)
*x* = 1.00	pyrochlore^a^	10.12655(17)				1038.448(53)

a Indicates phase purity.

**Table 2:** Average Composition of Zirconolite-2M, Zirconolite-4M, and Cubic Pyrochlore as Determined by Semiquantitative EDS Analysis (Normalized to Seven Oxygen Atoms)

	average composition from EDS		
nominal composition	zirconolite-2M	zirconolite-4M	pyrochlore
*x* = 0.10	Ca_0.91(4)_Zr_0.76(9)_Dy_0.17(4)_Ti_2.16(7)_O_7_		
*x* = 0.20	Ca_0.86(5)_Zr_0.69(8)_Dy_0.29(3)_Ti_2.16(7)_O_7_	Ca_0.76(9)_Zr_0.64(5)_Dy_0.52(7)_Ti_2.08(10)_O_7_	
*x* = 0.30	Ca_0.78(10)_Zr_0.78(9)_Dy_0.37(4)_Ti_2.07(9)_O_7_	Ca_0.71(2)_Zr_0.65(3)_Dy_0.54(3)_Ti_2.10(4)_O_7_	
*x* = 0.40		Ca_0.56(4)_Zr_0.59(7)_Dy_0.84(13)_Ti_2.01(15)_O_7_	Ca_0.48_Zr_0.41(5)_Dy_1.06(10)_Ti_2.08(9)_O_7_
*x* = 0.50		Ca_0.55(6)_Zr_0.72(3)_Dy_0.79(10)_Ti_2.14(7)_O_7_	Ca_0.46(6)_Zr_0.40(9)_Dy_1.11(9)_Ti_2.03(9)_O_7_
*x* = 0.60			Ca_0.39(5)_Zr_0.37(3)_Dy_1.09(6)_Ti_2.14(7)_O_7_
*x* = 0.70			Ca_0.29(1)_Zr_0.25(2)_Dy_1.35(8)_Ti_2.11(7)_O_7_
*x* = 0.80			Ca_0.18(3)_Zr_0.17(2)_Dy_1.51(11)_Ti_2.14(11)_O_7_
*x* = 0.90			Ca_0.10(2)_Zr_0.09(2)_Dy_1.72(7)_Ti_2.09(8)_O_7_
*x* = 1.00			Dy_1.86(13)_Ti_2.14(13)_O_7_

**Figure 5:**
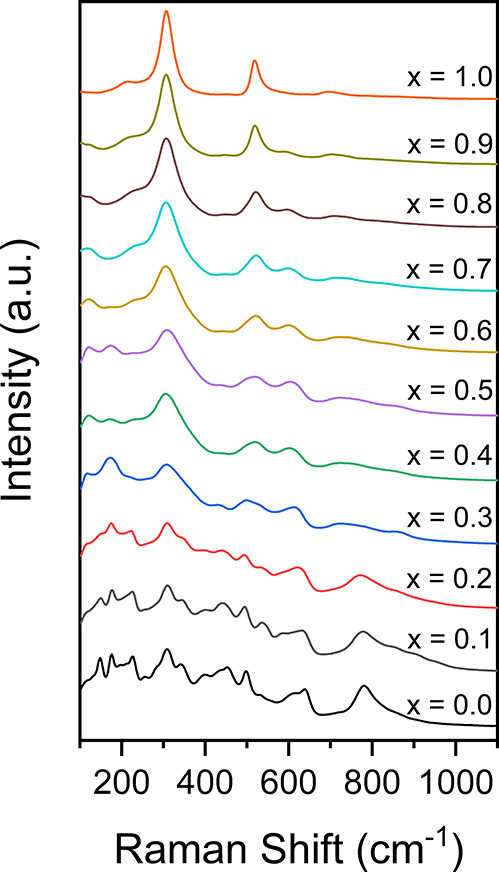
Raman spectra for Ca_1–*x*_Zr_1–*x*_Dy_2*x*_Ti_2_O_7_ in compositional range 0 ≤ *x* ≤ 1.00.

Further evidence of the structure transformation from zirconolite-2M to cubic pyrochlore was inferred by Raman data, collected in the range 100–1100 cm^–1^ (Figure 5). The position and intensity of vibrational modes for zirconolite-2M (*x* = 0.10) are in excellent agreement with our previous observations for nominal CaZrTi_2_O_7_ synthesized under identical conditions.^37^ The low symmetry of the monoclinic 2 M unit cell resulted in many active Raman vibrational modes. The dominant symmetric stretching vibration at 780 cm^–1^ was attributed to TiO_6_ octahedra.^25^ Raman active modes in the range 100–700 cm^–1^ have not been previously deconvoluted and assigned to individual vibrational modes; however, it is accepted that the spectrum consists of internal vibrations of the TiO_6_, CaO_8_, and ZrO_7_ polyhedral groups.^26^ As the nominal concentration of Dy^3+^ was increased for 0.10 ≤ *x* ≤ 0.40, a significant degree of broadening occurred in the spectral range 200–600 cm^–1^, attributed to the disorder induced by the accommodation of Dy^3+^ in the Ca^2+^ and Zr^4+^ sites, and associated polytype transformation to zirconolite-4M. A notable abatement of the dominant 780 cm^–1^ mode was also noted, an artifact we have recently observed in the Raman spectra of the CaZr_1–*x*_Ce*_x_*Ti_2_O_7_ zirconolite solid solution, for which a similar polytype transformation from zirconolite-2M to zirconolite-4M also occurred.^38^ Factor group analysis has previously determined that the general cubic pyrochlore structure A_2_B_2_O(1)_6_O(2) gives rise to six Raman active vibrational modes: A_g_, E_g_, and 4F_2g_.^39^ A reasonable fit for the end member Dy_2_Ti_2_O_7_ was achieved using a combination of pseudo-Voigt profile functions (Figure S1). Six modes were deconvoluted, corresponding to approximate wavenumbers: 206, 306, 331, 517, 547, and 697 cm^–1^. Bands for the Dy_2_Ti_2_O_7_ spectrum have also been assigned according to calculated wavenumbers for A_2_Ti_2_O_7_ pyrochlores (A = Y, Sm, Gd, Yb) by Gupta et al., accounting for slight variations in position, determined by *r*_A_/*r*_Ti_.^40^ The acquired Raman spectra are in excellent agreement with Y_2_Ti_2_O_7_, Gd_2_Ti_2_O_7_, Sm_2_Ti_2_O_7_, and Dy_2_Ti_2_O_7_ specimens formed by similar processing routes.^28,41,42^

### 3.2 Phase Field Confirmation by High-Resolution Transmission Electron Microscopy

**Figure 6:**
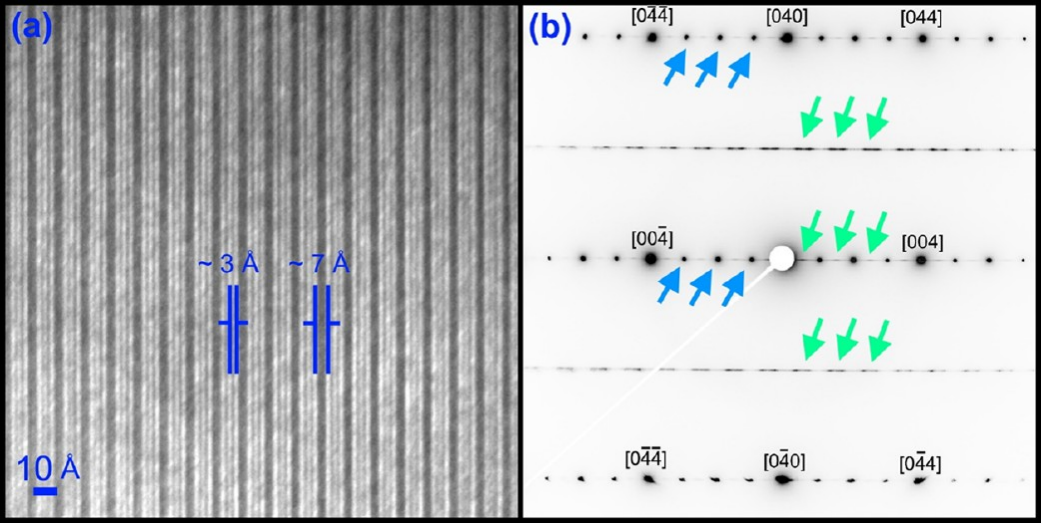
(a) Bright-field TEM micrograph of the *x* = 0.3 sample, showing the zirconolite-4M structure, with the electron beam positioned down the [100] zone axis and (b) a [100] zone-axis electron diffraction pattern indexed to the zirconolite-4M structure. Streaked reflections are identified by left-pointing green arrows.

**Figure 7:**
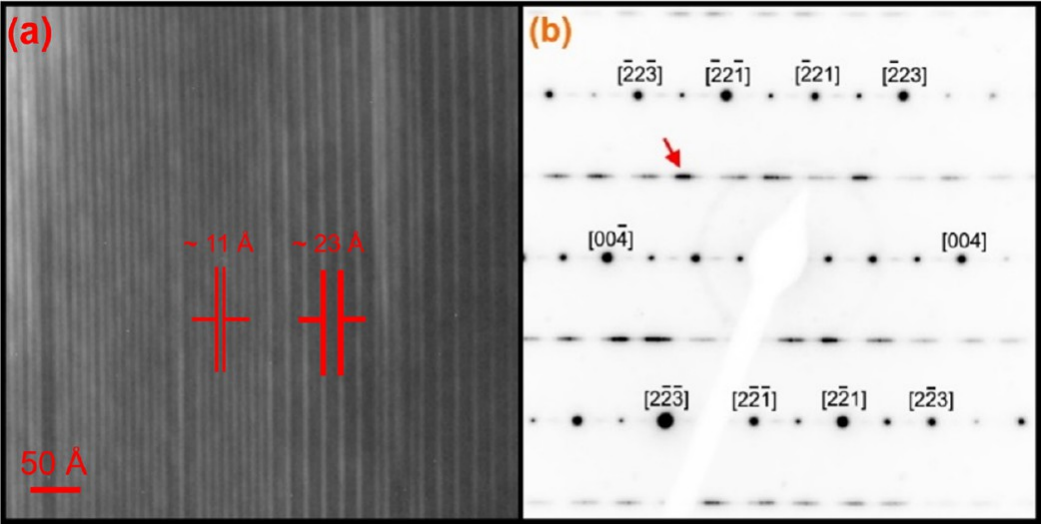
(a) Dark-field TEM micrograph of the *x* = 0.3 sample, showing the zirconolite-4M structure, with the electron beam positioned down the [110] zone axis and the objective aperture over the diffuse reflection indicated by the arrow in (b) a [110] zone-axis electron diffraction pattern indexed to the zirconolite-4M structure.

**Figure 8:**
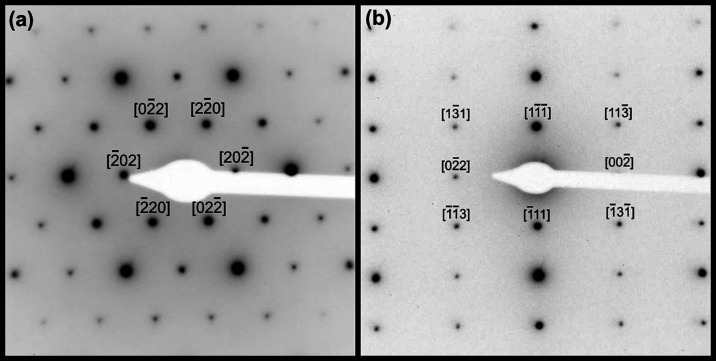
Zone-axis electron diffraction patterns of the *x* = 0.6 sample with the electron beam positioned down the (a) [111] and (b) [211] zone axes. Both patterns are indexed to the pyrochlore *Fd*3̅*m* structure.

The phase fields identified by powder XRD and SEM analyses were further evidenced by high-resolution transmission electron (HR-TEM) microscopy, coupled with selected area electron diffraction (SAED). As electron diffraction analysis allows variations in stacking sequence to be distinguished, polymorphic transitions in REE^3+^/Ac^4+^-doped zirconolites can be reconciled. As presented in Figure 6a, high-resolution TEM analysis of the *x* = 0.3 composition shows the layered structure of zirconolite-4M, as has been previously reported.^16^ The bright-field HRTEM image presented in Figure 6a was captured with the beam orientated down the [110] zone axis, as is shown by the indexed electron diffraction pattern in Figure 6b. “Streaked” reflections can be observed in Figure 6b, as identified by the left-pointing green arrows, and clearly discernible ordered reflections, identified with right-pointing blue arrows. Such reflections are the direct consequence of the stacking sequence observed in Figure 6a, induced through the varied layer spacings indicated in this micrograph (*i.e.*, ∼3 and ∼7 Å). This area shows a highly ordered stacking sequence in what is a grain of the zirconolite-4M structure, leading to the strong reflections observed, while the varied layer spacings produced the “streaked” reflections. The reduced contrast is likely induced by a layer rich in Dy, similar to Nd-doped zirconolite studies that have been reported, although high-angle annular dark-field imaging would be required to confirm this hypothesis. The dark-field micrograph presented in Figure 7a shows a two-layered band structure down the [110] zone axis, imaged with the objective aperture positioned over the diffuse reflection indicated by the right-pointing red arrow. The two-layered bands have spacings of ∼23 and ∼11 Å, representing a doubling of the unit cell along the *c*-axis. As described by Coelho et al., these imperfections are commonplace throughout an indexed zirconolite-4M structure and indicate the presence of both 4M and 2M spacings within a single-crystal grain. In contrast to Figure 6, this area contained nonuniform domains of varied spacing, suggesting variations in the level of 4M ordering within each crystal for the *x* = 0.30 sample. Analysis of the *x* = 0.60 composition through electron diffraction confirmed the formation of the pyrochlore structure, as presented in Figure 8a,b for the [111] and [211] zone axis patterns, respectively. No evidence of a zirconolite-2M or 4M phase was detected throughout the grains observed, confirming the phase transition to a pure pyrochlore phase at *x* = 0.60, in agreement with powder XRD and SEM observations.

### 3.3 XAS Investigations of Dy Oxidation State and Coordination

**Figure 9:**
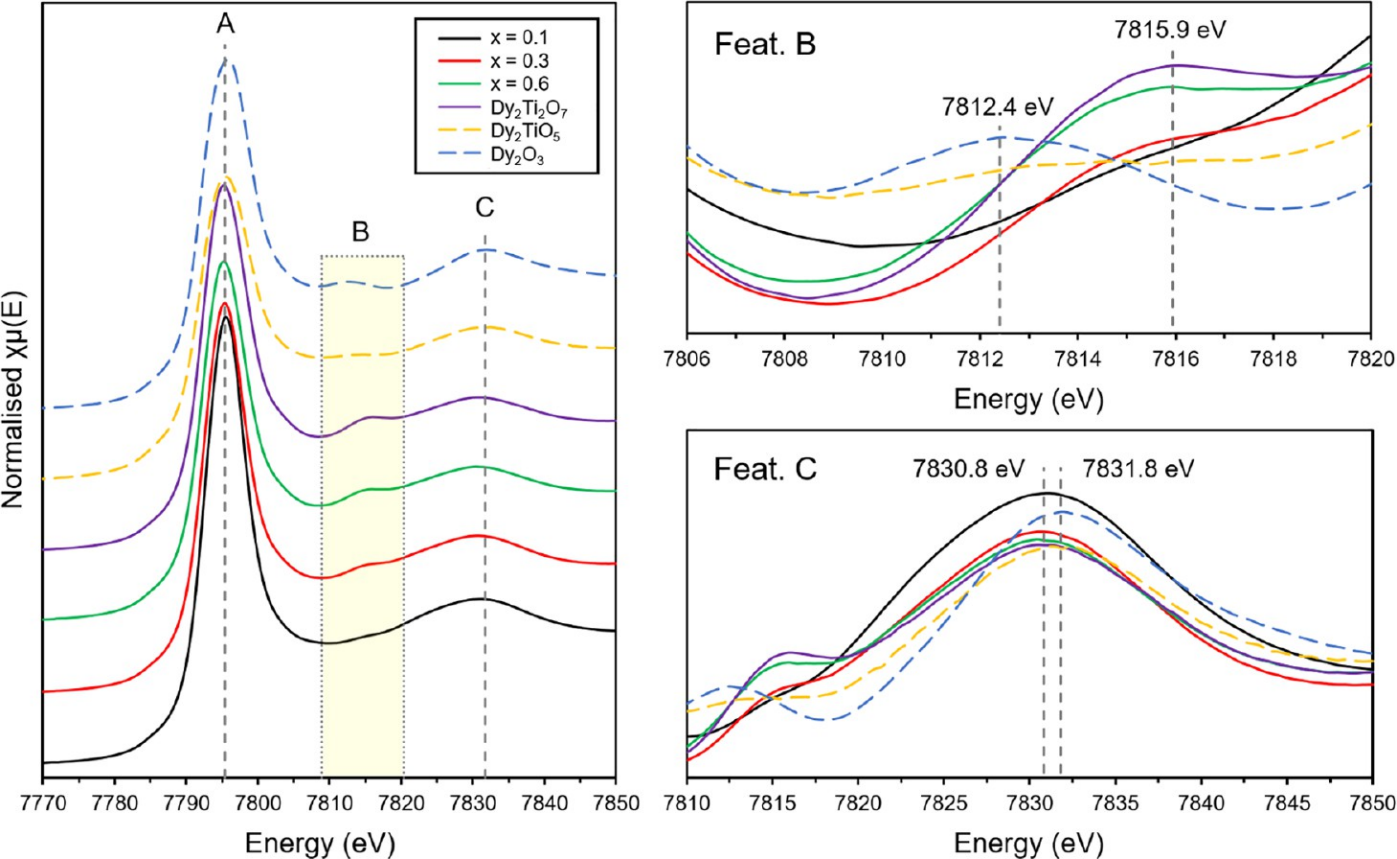
XANES data collected for *x* = 0.10, *x* = 0.30, and *x* = 0.60, alongside Dy_2_O_3_, Dy_2_TiO_5_, and Dy_2_Ti_2_O_7_ reference compounds, with features **A**, **B**, and **C** labeled. Insets on the right show enlargements of features **B** and **C**.

Dy L_3_-edge X-ray absorption near edge structure (XANES) spectra were collected for zirconolite-2M, zirconolite-4M, and cubic pyrochlore (corresponding to *x* = 0.10, 0.30, and 0.60, herein referred to as Dy-titanates for clarity) alongside Dy_2_O_3_, Dy_2_TiO_5_, and Dy_2_Ti_2_O_7_ reference compounds, containing Dy^3+^ in 6-, 7-, and 8-fold coordination, respectively (Figure 9). Experimental XANES spectra at the Dy L_3_-edge of all reference compounds and Dy-titanates were characterized by three distinct features (labeled as **A**, **B**, and **C** in Figure 9 and Table S2). Primarily, the white line crest (feature **A**) was composed of a single intense feature for all compounds at the overlapping edge position of 7792.5 ± 0.3 eV. The major contribution to this feature arises from dipole allowed 2p_3/2_ → 5d_3/2_ electronic transitions.^43^ Theoretically, absorption spectra at the Dy L_3_-edge also comprise a weak pre-edge feature; however, this cannot be resolved by conventional XAS due to 2p core-hole lifetime broadening. Nevertheless, this feature can be observed with complementary techniques such as resonant inelastic scattering spectroscopy (RIXS).^44^ Second, a weak yet discernible feature (labeled as **B** in Figure 9 and Table S2) was also clearly distinguished (we note that this was most prominent in Dy_2_Ti_2_O_7_, Dy_2_O_3_, and the sample corresponding to *x* = 0.60). Finally, a post-edge resonance peak (feature **C** in Figure 9 and Table S2) was observed for all compounds, with maxima between 7830.8 and 7831.8 eV.

There were several qualitative trends noted in the XANES spectra of the reference compounds and Dy-titanates. Primarily, it was clear that the edge position (7792.5 ± 0.3 eV) and energy position of feature **A** were very similar for all reference compounds and Dy-titanates, indicating that all samples contained Dy in the same oxidation state. As the reference compounds all contained Dy uniformly as Dy^3+^, it was therefore considered that Dy entered the zirconolite-pyrochlore solid solution entirely as Dy^3+^. Further confidence in Dy oxidation state assignment was informed from bond valence sum analyses, the results of which are summarized in Table 3. The speciation of Dy^3+^ is encouraging, as this remains comparable to Am^3+^; previously, the synthesis of Am_2_Ti_2_O_7_ by calcination in air between 1200 and 1300 °C has been shown to result in the complete reduction of Am^4+^ to Am^3+^.^45^ Feature **B** presented a variation that may be dependent on the coordination of Dy^3+^ cations. This is evidenced by the reference compounds (Dy_2_TiO_5_ and Dy_2_O_3_) having different energy maxima (∼7812.4 eV) compared to the Dy-titanates *x* = 0.10, 0.30, 0.60, and 1.00 (Dy_2_Ti_2_O_7_) (∼7815.9 eV) (Figure 9 and Table S2). This shift in maxima position could be attributed to an increase in O coordination of the Dy^3+^ atoms, as the feature maximum for the 6-fold Dy_2_O_3_ is lower than that of the 8-fold Dy_2_Ti_2_O_7_; however, a more comprehensive systematic analysis of L_1_- and L_2_-edges would be needed to confirm this trend. Moreover, the intensity of feature **B** present in Dy-titanates was also observed to vary as a function of Dy concentration and thus changing structure type. This can clearly be seen when comparing feature **B** intensity between zirconolite-2M (*i.e.*, *x* = 0.10) in which Dy^3+^ was targeted equimolar across both the 8- and 7-fold sites, and pyrochlore (*i.e.*, *x* = 0.60) in which Dy^3+^ cations occupy only one 8-fold coordinated site. This is qualitative evidence that suggests that there is an agreement between the targeted self-balancing charge substitution scheme for zirconolite and Dy^3+^ being split between two crystallographically distinct sites at a lower concentration. Qualitative trends were also observed for feature **C**, in which a decrease in maxima (7830.8 eV) intensity was observed in correlation with increased Dy doping of the Dy-titanates. Additionally, a shift in maxima position of ∼0.9 eV is seen between the Dy-titanate samples and the reference compounds (Dy_2_O_3_ and Dy_2_TiO_5_) possibly indicating a change in Dy coordination environment. A similar coordination-related energy shift in this feature has also been noted in the Dy and Sm L_3_-edges for other complex materials and has been proposed as the result of increased average Ln–O bond distance.^43,46^ This qualitative trend is broadly consistent with EXAFS analyses (discussed below) whereby a slight decrease in the average Dy–O bonds was observed with increasing Dy concentration (*i.e.*, when Dy was modeled as the absorbing atom in zirconolite-2M, 4M, and pyrochlore).

**Table 3:** Bond Valence Sums for Dy in Selected Compositions

composition	bond valence sum (Dy···O x8)
*x* = 0.10 (zirconolite-2M)	3.125
*x* = 0.30 (zirconolite-4M)	2.98
*x* = 0.60 (pyrochlore)	3.021
*x* = 1.00 (pyrochlore)	3.016

**Figure 10:**
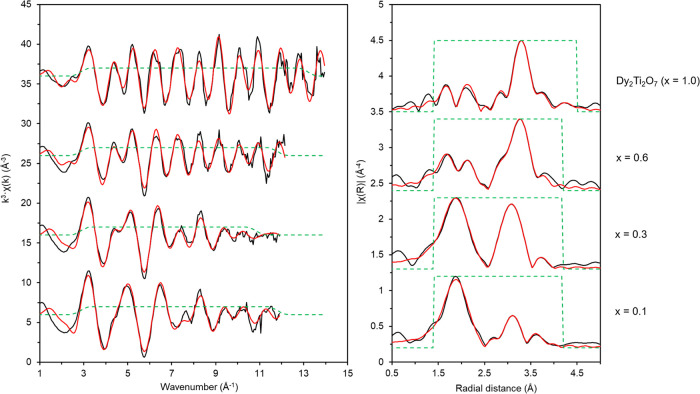
Dy L_3_ edge XAS spectra for Dy_2_Ti_2_O_7_ (*i.e.*, *x* = 1.0), *x* = 0.6, *x* = 0.3, and *x* = 0.1 samples (where x refers to the structure Ca_1–*x*_Zr_1–*x*_Dy_2*x*_Ti_2_O_7_). Left: *k*^3^-weighted EXAFS. Right: Fourier transform of *k*^3^-weighted EXAFS, using a Hanning window function. Black lines are data, and red lines are the best modeled fits for the data.

Fitting of the EXAFS region provided insight into the local structure of Dy^3+^ including the immediate coordination environment and structure over a range of up to ∼4.5 Å from the central Dy atom (Figure 10; Table 4). Analysis of the Dy_2_Ti_2_O_7_ reference compound (*i.e.*, *x* = 1.00) produced a good fit (*R*-factor = 0.0155) that consisted of 2 O atoms at 2.19(2) Å, 6 O atoms at 2.45(2) Å, 6 Ti atoms at 3.57(1) Å, 6 Dy atoms at 3.58(1) Å, 12 O atoms at 3.92(5) Å, 6 O atoms at 4.51 Å, and an O–Ti multiple scattering pathway at 4.79 Å with a degeneracy of 24. This model is in excellent agreement with the expected Dy_2_Ti_2_O_7_ structure as previously determined by single-crystal X-ray diffraction.^34^ Fitting of the Dy_2_Ti_2_O_7_ reference compound informed the fitting parameters of the *x* = 0.60 compound, as from XRD and TEM analyses, this was confirmed to also adopt the cubic pyrochlore structure, despite partial occupancy of Ca and Zr on the A site. An excellent fit (*R*-factor = 0.0076) to the data was produced with a similar model to the Dy_2_Ti_2_O_7_ standard, albeit with a lower coordination of Dy^3+^ atoms and fewer long-range order shells fitted. The best-fit model consisted of 2 O atoms at 2.21(2) Å, 6 O atoms at 2.43(2) Å, 6 Ti atoms at 3.53(1) Å, 3.6 Dy atoms at 3.59(2) Å, and 12 O atoms at 3.93(5) Å. The number of second shell Dy···Dy paths was reduced from 6 to 3.6 (*x* = 1.00 and 0.60, respectively), which is in line with the reduced concentration of Dy in the *x* = 0.60 sample relative to the *x* = 1.00 (Dy_2_Ti_2_O_7_) compound. As the coordination shell only contains 3.6 Dy, this leaves a remaining degeneracy of 2.4 that is made up of Ca and Zr; however, attempts to fit these atoms into the EXAFS fits proved unsuccessful, with the addition resulting in significantly worse fits (see Supporting Information for further details). This may be due to the limited data obtained of the samples (*k*-range could only be fit to 12), and therefore, a much higher k-range may be needed to deconvolute the Ti, Dy, Ca, and Zr that all manifest at a similar point in the EXAFS spectrum. Indeed, such limitations have been observed previously in different systems with different elements (U in iron (oxyhydr)oxides); however, the need for a high *k*-range, and the addition of molecular dynamics modeling, to deconvolute many overlapping shells in the EXAFS has been clearly shown.^47,48^

**Table 4:** Fitting Parameters for EXAFS Data Presented in Figure 10^a^

		path								
sample	parameters	O1	O2	Ti1	Ti2	Zr1	Dy1	O3	O4	O Ti MS
	*N*	2	6	6			6	12	6	24
Dy_2_Ti_2_O_7_ (*x* = 1.0)	σ^2^(10^–3^) (Å^2^)	3(1)	13(1)	13(2)			7(1)	15(7)	5(4)	9^b^
*E*_0_ = 1.4(14)	*R*(Å)	2.19(2)	2.45(2)	3.57(1)			3.58(1)	3.92(5)	4.51(3)	4.79^b^
*R*-factor = 0.0155	α(%)	100.0	100.0	100.0			100.0	100.0	99.7	
	*N*	2	6	6			3.6	12		
*x* = 0.6	σ^2^(10^–3^) (Å^2^)	7(2)	15(2)	15(2)			7(1)	19(7)		
*E*_0_ = 1.6(13)	*R*(Å)	2.21(2)	2.43(2)	3.53(1)			3.59(2)	3.93(5)		
*R*-factor = 0.0076	α(%)	99.9	100.0	100.0			99.4	100.0		
	*N*	5	3	6			0.6	8		
*x* = 0.3	σ^2^(10^–3^) (Å^2^)	12(4)^c^	12(4)^c^	13(1)			4(3)	18(4)		
*E*_0_ = 2.6(6)	*R*(Å)	2.29(5)	2.41(2)	3.49(1)			3.59(4)	4.05(4)		
*R*-factor = 0.0028	α(%)	100.0	100.0	100.0			94.6	100.0		
	*N*	4	4	2	4	1		12		
*x* = 0.1	σ^2^(10^–3^) (Å^2^)	5(1)^c^	5(1)^c^	9(7)^d^	9(7)^d^	7(3)		19(3)		
*E*_0_ = 3.2(5)	*R*(Å)	2.28(1)	2.43(1)	3.33(3)	3.52(2)	3.58(10)		4.13(2)		
*R*-factor = 0.0040	σ(%)	100.0	100.0	94.0	99.4	100.0		99.9		

a The amplitude reduction factor (*S*_0_^2^) for all samples was 0.95; *N* is the degeneracy; σ^2^ is the Debye–Waller factor; *R* is the interatomic distance; α is the result of the F-test indicating the confidence that adding the path improves the fit (>67% is equal to 1σ and >95 % is equal to 2σ in terms of standard deviation).

b Indicates that the path was parameterized using O1 and Ti1 parameters.

c Indicates that the parameters were linked.

d Indicates that the parameters were linked. The general formula relating to “*x*” is Ca_1–*x*_Zr_1–*x*_,Dy_2*x*_Ti_2_O_7_.

Fitting of the EXAFS for the *x* = 0.1 sample to a zirconolite-2M model produced a good fit (*R*-factor = 0.0040) with the model consisting of 4 O atoms at 2.28(1) Å, 4 O atoms at 2.43(1) Å, 2 Ti atoms at 3.33(3) Å, 4 Ti atoms at 3.52(2) Å, 1 Zr at 3.58(10) Å, and 12 O at 4.13 Å. This is in agreement with the expected zirconolite-2M structure. The *x* = 0.3 sample proved the most challenging to fit, due to the composition adopting the complex zirconolite-4M structure. The zirconolite-4M structure is composed of both pyrochlore and zirconolite-2M structural units, resulting in a system of mixed coordination environments for the Dy^3+^ ions. Consequently, a fit that represented a mixture of the zirconolite-2M and pyrochlore structure was attempted, using a pyrochlore CIF file as the basis. A good fit was obtained (*R*-factor = 0.0028) which consisted of 5 O atoms at 2.29(5) Å, 3 O atoms at 2.41(2) Å, 6 Ti atoms at 3.49(1) Å, 0.6 Dy atoms at 3.59(4) Å, and 8 O atoms at 4.05(4) Å. The reduced occupancy of the distal O shell (8 instead of 12) is likely representative of the lower degree of long-range order in the structure. The degeneracy of the Dy shell is concordant with expected occupancy of the zirconolite-2M structure. As with the previous samples and as discussed above, the Ca and Zr atoms could not be fit due to the limited *k*-range obtained.

While EXAFS fitting suggests that the Dy^3+^ cations are 8-fold coordinated by O atoms in all compositions, this coordination environment is only expected to be the sole environment for the Dy_2_Ti_2_O_7_ pyrochlore reference compound. Upon doping the zirconolite-2M structure with Dy^3+^, it is expected that the Dy atoms will occupy the Ca (8-fold) and Zr (7-fold) sites, with a possible preference for the Ca site on the basis of compatible ionic radii (Ca^2+^ = 1.12 Å and Dy^3+^ = 1.027 Å in 8-fold coordination).^49^ In the *x* = 0.1 sample, an even split of 4 O atoms at 2.28(1) Å (Zr site) and 4 O atoms 2.43(1) Å (Ca site) may suggest that Dy is equally distributed across the Ca (8-fold) and Zr (7-fold) sites. Indeed, altering the total occupancy of the first O shell to 7.5 (expected in a system with a 50:50 split of 7- and 8-fold sites) by setting the degeneracy of the aforementioned O atoms to 3.75 each produces an equally valid fit, albeit with a slightly worsened R-factor of 0.0073, with a similar effect being observed in the *x* = 0.3 composition (increased *R*-factor of 0.0032). This suggests that Dy^3+^ may be doped in equal amounts across the Ca and Zr sites, in good agreement with the targeted self-charge balancing substitution regime, and moreover, the qualitative observations of features **B** and **C** in the XANES region.

### 3.4 Discussion within Context of Existing Literature

**Figure 11:**
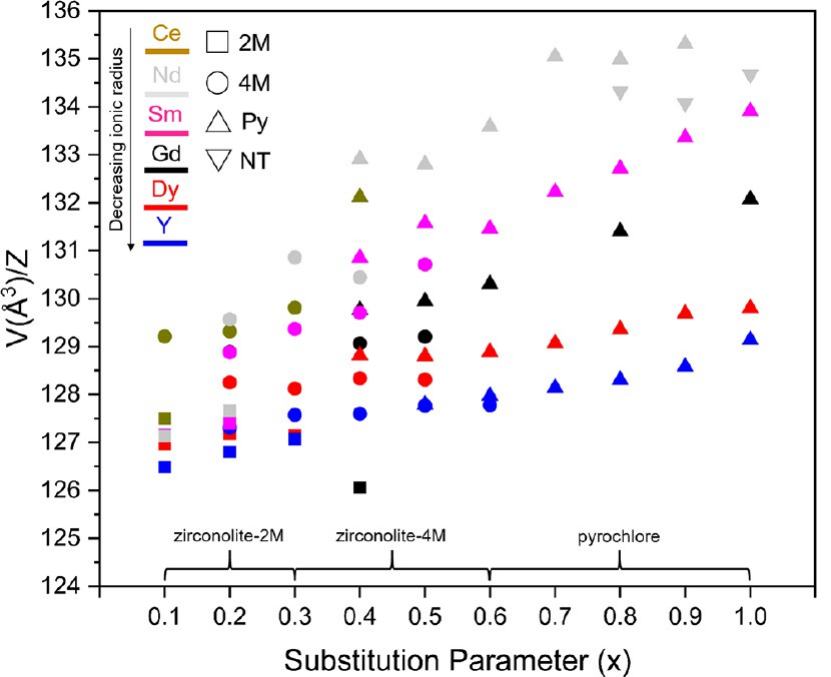
Visual summary of phase fields reported in CaZrTi_2_O_7_–REE^3+^Ti_2_O_7_ solid solutions with nominal formulation Ca_1–*x*_Zr_1–*x*_REE_2*x*_Ti_2_O_7_ (Key: 2M = zirconolite-2M; 4M = zirconolite-4M; Py = cubic pyrochlore; NT = Nd_2_Ti_2_O_7_ (monoclinic double-layered perovskite, space group *P*2_1_)).

Several systematic CaZrTi_2_O_7_–REE^3+^Ti_2_O_7_ solid solutions have been reported in the wider literature, the published phase fields from which are summarized in Table 5 and Figure 11, with descriptions below. A comparison of the ionic radii of lanthanides and minor actinides is provided in Table S1. The radius ratio necessary to stabilize the cubic pyrochlore structure in A_2_B_2_O_7_ ceramics is clearly demonstrated by varying the size of the A^3+^ site, in agreement with the model proposed by McCauley.^50^ The bracketed areas displayed along the x-axis in Figure 11 represent the dominant phase present at a given substitution value.

**Table 5:** Summary of Reported Phase Fields for CaZrTi_2_O_7_–REE^3+^Ti_2_O_7_ in Order of Increasing Ionic Radius (8-Fold Coordination), Where REE^3+^ = Y, Dy, Gd, Sm, Nd, and Ce^a^

		phase assemblage									
solid solution	ionic radius (Å)	*x* = 0.10	*x* = 0.20	*x* = 0.30	*x* = 0.40	*x* = 0.50	*x* = 0.60	*x* = 0.70	*x* = 0.80	*x* = 0.90	*x* = 1.00
Ca_1–*x*_Zr_1–*x*_Y_2*x*_Ti_2_O_7_	1.019	2M^b^	2M + 4M	2M + 4M	4M^b^	4M + Py	4M + Py	Py^b^	Py^b^	Py^b^	Py^b^
Ca_1–*x*_Zr_1–*x*_Dy_2*x*_Ti_2_O_7_	1.027	2M^b^	2M + 4M	2M + 4M	4M + Py	4M + Py	Py^b^	Py^b^	Py^b^	Py^b^	Py^b^
Ca_1–*x*_Zr_1–*x*_Gd_2*x*_Ti_2_O_7_	1.053	2M^b^	2M + 4M + Pe		2M + 4M + Pe + Py	4M + Py	Py^b^		Py^b^		Py^b^
Ca_1–*x*_Zr_1–*x*_Sm_2*x*_Ti_2_O_7_	1.079	2M + Pe	2M + 4M + Pe	4M + Pe	4M + Py	4M + Py	Py^b^	Py^b^	Py^b^	Py^b^	Py^b^
Ca_1–*x*_Zr_1–*x*_Nd_2*x*_Ti_2_O_7_	1.109	2M + Pe	2M + 4M + Pe	4M + Pe	4M + Pe + Py	Py + Pe	Py + Pe	Py + Pe	Py + Pe + NT	NT^b^	NT^b^
Ca_1–*x*_Zr_1–*x*_Ce_2*x*_Ti_2_O_7+δ_	1.143	2M + 4M + Pe	4M^b^	4M^b^	Py + Pe						

a Key: 2M = zirconolite-2M; 4M = zirconolite-4M; Py = cubic pyrochlore; Pe = perovskite; NT = Nd_2_Ti_2_O_7_ (monoclinic double-layered perovskite, space group *P*2_1_).

b Indicates phase purity.

**Gd**^**3+**^: Zhang et al. synthesized the Ca_1–*x*_Zr_1–*x*_Gd_2*x*_Ti_2_O_7_ solid solution (0.0 ≤ *x* ≤ 1.0) by oxide synthesis, with bulk pellets sintered at 1400 °C for 48 h.^25^ The zirconolite-2M phase was present over the compositional range 0.0 ≤ *x* ≤ 0.4, while the 4M polytype was stabilized between 0.2 ≤ *x* ≤ 0.5. The cubic pyrochlore phase was formed in compositions for which *x* ≥ 0.4, and present as a single phase above *x* = 0.6. These phase fields are near-identical to those reported in the present work, which is unsurprising given the relative ionic radii of Gd^3+^ and Dy^3+^ cations (1.053 and 1.027 Å, respectively).

**Sm**^**3+**^: Similar phase fields were reported by Jafar et al. in the Ca_1–*x*_Zr_1–*x*_Sm_2*x*_Ti_2_O_7_ (0.0 ≤ *x* ≤ 1.0) system, fabricated by solid-state reaction at 1300 °C (24 h).^28^ Zirconolite-2M was found to coexist alongside a minor perovskite phase when targeting *x* = 0.10, with mixtures of zirconolite-2M, 4M, and/or perovskite formed in the compositional range 0.20 ≤ *x* ≤ 0.35. Cubic pyrochlore and zirconolite-4M were reported for *x* = 0.40 and 0.50, with single-phase pyrochlore observed beyond *x* ≥ 0.60.

**Y**^**3+**^: Jafar et al. also synthesized corresponding Ca_1–*x*_Zr_1–*x*_Y_2*x*_Ti_2_O_7_ compositions with a sintering temperature of 1300 °C.^26^ No perovskite phases were identified at any interval, with single phase 2M and 4M formed at *x* = 0.10 and 0.40, respectively, with the intermediate compositions composed of a mixture of polytypes. At *x* = 0.60, zirconolite-4M was found to coexist with pyrochlore; however, cubic pyrochlore was stabilized as a single phase beyond *x* ≥ 0.70.

**Nd**^**3+**^: The phase fields observed when doping Nd^3+^ into the structure were observed to deviate from the conventional 2M → 4M → Py phase evolution, as zirconolite-2M, 4M, or cubic pyrochlore was not formed as a single phase at any compositional interval.^51^ Rather, co-mixtures of 2M, 4M, and/or perovskite were present between 0.10 ≤ *x* ≤ 0.40, progressing to mixtures of cubic pyrochlore and perovskite between 0.50 ≤ *x* ≤ 0.70. As the end-member Nd_2_Ti_2_O_7_ structure is not capable of adopting the cubic pyrochlore structure on the basis of ionic radii (Nd^3+^ = 1.109 Å), a monoclinic double-layered perovskite structure was preferentially formed beyond *x* = 0.90.

**Ce**^**3+**^: Meng et al. synthesized the corresponding Ce solid solution; however, as Ce is known to adopt both Ce^3+^ and Ce^4+^ oxidation states in zirconolite, the targeted composition allowed for nonideal oxygen stoichiometry, and thus denoted as Ca_1–*x*_Zr_1–*x*_Ce_2*x*_Ti_2_O_7+δ_.^4^ Moreover, Ce 3d XPS data confirmed that Ce was consistently distributed as ∼50% Ce^3+^, inferring that the excess positive charge could be compensated by cation vacancies. Unfortunately, the solid solution was not progressed beyond *x* = 0.40, but, given the relatively large radius of Ce^3+^ in comparison to other REE^3+^ cations, the formation of single-phase zirconolite-4M was detected at a lower concentration, corresponding to both *x* = 0.20 and 0.30. When targeting *x* = 0.40, a co-mixture of pyrochlore and perovskite was formed.

## 5 Conclusions

Zirconolite-structured materials are a candidate wasteform for the immobilization of Pu and other highly radioactive minor actinide species that may be derived from future advanced reprocessing cycles for spent nuclear fuel. To this end, the novel Ca_1–*x*_Zr_1–*x*_Dy_2*x*_Ti_2_O_7_ solid solution was fabricated by a conventional solid-state route, with Dy^3+^ deployed as an inactive surrogate cation to replicate the partitioning behavior of minor actinides such as Am^3+^. XRD, SEM, TEM-ED, and XAS techniques were used to characterize a series of distinct phase transformations, with Dy^3+^ cations fully immobilized in the zirconolite-2M phase at a concentration corresponding to *x* = 0.10, followed by progressive mixtures of zirconolite-2M, 4M, and/or pyrochlore in the compositional interval 0.20 ≤ *x* ≤ 0.50. Increasing the nominal Dy^3+^ concentration beyond *x* = 0.60 resulted in the formation of single-phase pyrochlore, successfully forming the end-member Dy_2_Ti_2_O_7_. Analyses of the Dy L_3_ XANES and EXAFS regions confirm uniform Dy^3+^ speciation, consistent with previously observed Am^3+^ under similar processing conditions, and determine that the coordination environment of Dy cations was consistent with occupation in zirconolite-2M, zirconolite-4M, and pyrochlore-structures when targeting *x* = 0.10, 0.30, and 0.60, respectively. Given the exceptional radiation stability and chemical durability of zirconolite and pyrochlore solid solutions, it is expected that trivalent minor actinide species could be successfully accommodated in solid solution at any compositional interval in the CaZrTi_2_O_7_–Dy_2_Ti_2_O_7_ system.

## Supporting Information Available

Deconvolution of Raman spectrum obtained for Dy_2_Ti_2_O_7_ (Figure S1); deconvoluted EXAFS spectra for all samples (Figure S2); ionic radii for relevant lanthanides and minor actinides (Table S1); XANES features from Figure 9 and corresponding energies (Table S2); and EXAFS fitting parameters for a range of fitting models (Table S3) (PDF)
