# Supplementary material for: Phase Evolution in the CaZrTi2O7–Dy2Ti2O7 System: A Potential Host Phase for Minor Actinide Immobilization
Source: Inorg Chem. 2022 Apr 4;61(15):5744–56. doi: 10.1021/acs.inorgchem.1c03816 (PMC9019813; doi:10.1021/acs.inorgchem.1c03816)
Supplement: Supplementary file 1 — ic1c03816_si_001.pdf [file ic1c03816_si_001.pdf]

Supplementary Information for "Phase Evolution in the $CaZrTi_2O_7$ – $Dy_2Ti_2O_7$ System: A Potential Host Phase for Minor Actinide Immobilisation"

Lewis R. Blackburn[a], Luke Townsend[a], Sebastian M. Lawson[a,b], Amber R. Mason[a], Martin C. Stennett[a], Shi-Kuan Sun[a,c], Laura J. Gardner[a], Ewan R. Maddrell[d], Claire L. Corkhill[a], and Neil C. Hyatt[a]

[a]Immobilisation Science Laboratory, University of Sheffield, Department of Materials Science and Engineering, Sir Robert Hadfield Building, Mappin Street, S1 3JD, UK

[b]GeoRoc International (GRI) Ltd, Whitehaven, Cumbria, CA28 8PF, UK

[c]School of Materials Science and Energy Engineering, Foshan University, Foshan 528000, China

[d]National Nuclear Laboratory, Workington, Cumbria, CA20 1PJ, UK

Corresponding author: lewis.blackburn@sheffield.ac.uk

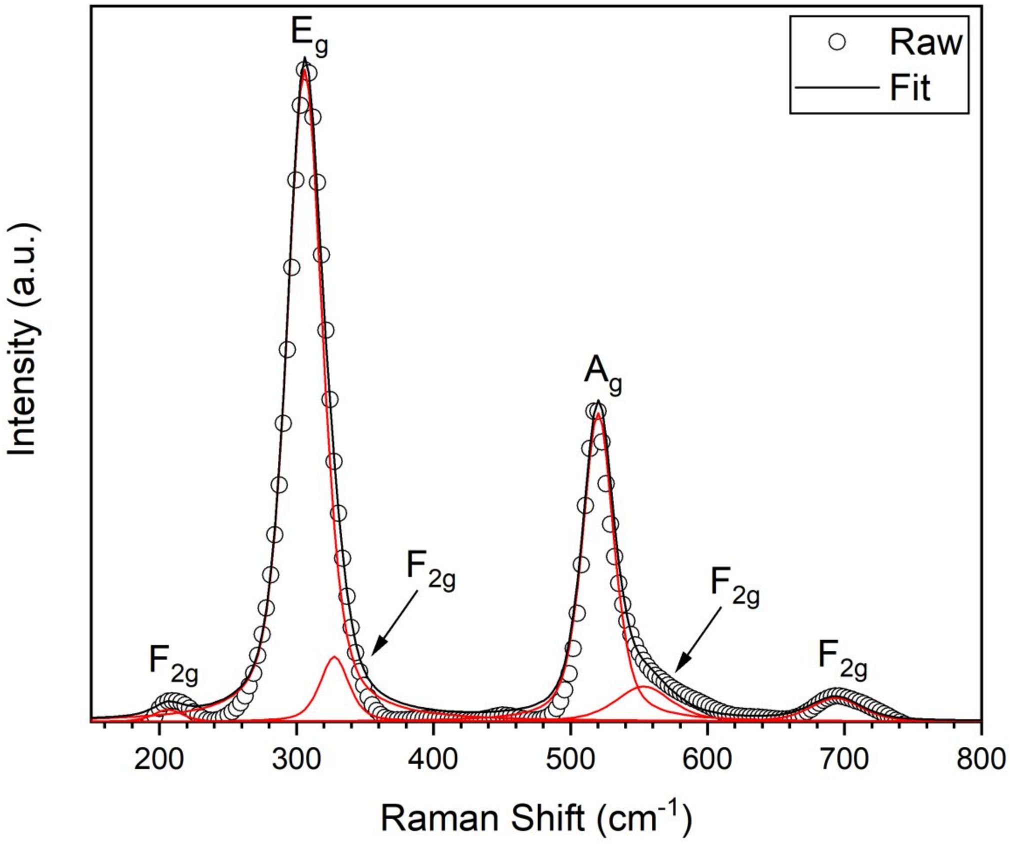

Fig. S1) Deconvolution of Raman spectrum obtained for $Dy_2Ti_2O_7$

Table S1) Ionic radii for relevant lanthanides and minor actinides to allow comparison with **Table 5**. Note that, due to data availability, all radii are quoted for 6-fold coordination.

Lanthanide	Ionic Radius (Å)	Minor actinide	Ionic Radius (Å)
Ce^{3+}	1.01	Np^{3+}	1.01
Nd^{3+}	0.983	Pu^{3+}	1.00
Sm^{3+}	0.958	Am^{3+}	0.975
Gd^{3+}	0.938	Cm^{3+}	0.97
Tb^{3+}	0.923	Bk^{3+}	0.96
Dy^{3+}	0.912	Cf^{3+}	0.95

Table S2) XANES features from Fig. 9 and corresponding energies. The edge position is given as an average value with its associated standard deviation

**Feature**	**Energy Position (eV)**
Edge Position	7792.5 ± 0.3
A	7795.4
B	7812.4 – 7815.9
C	7830.8 – 7831.8

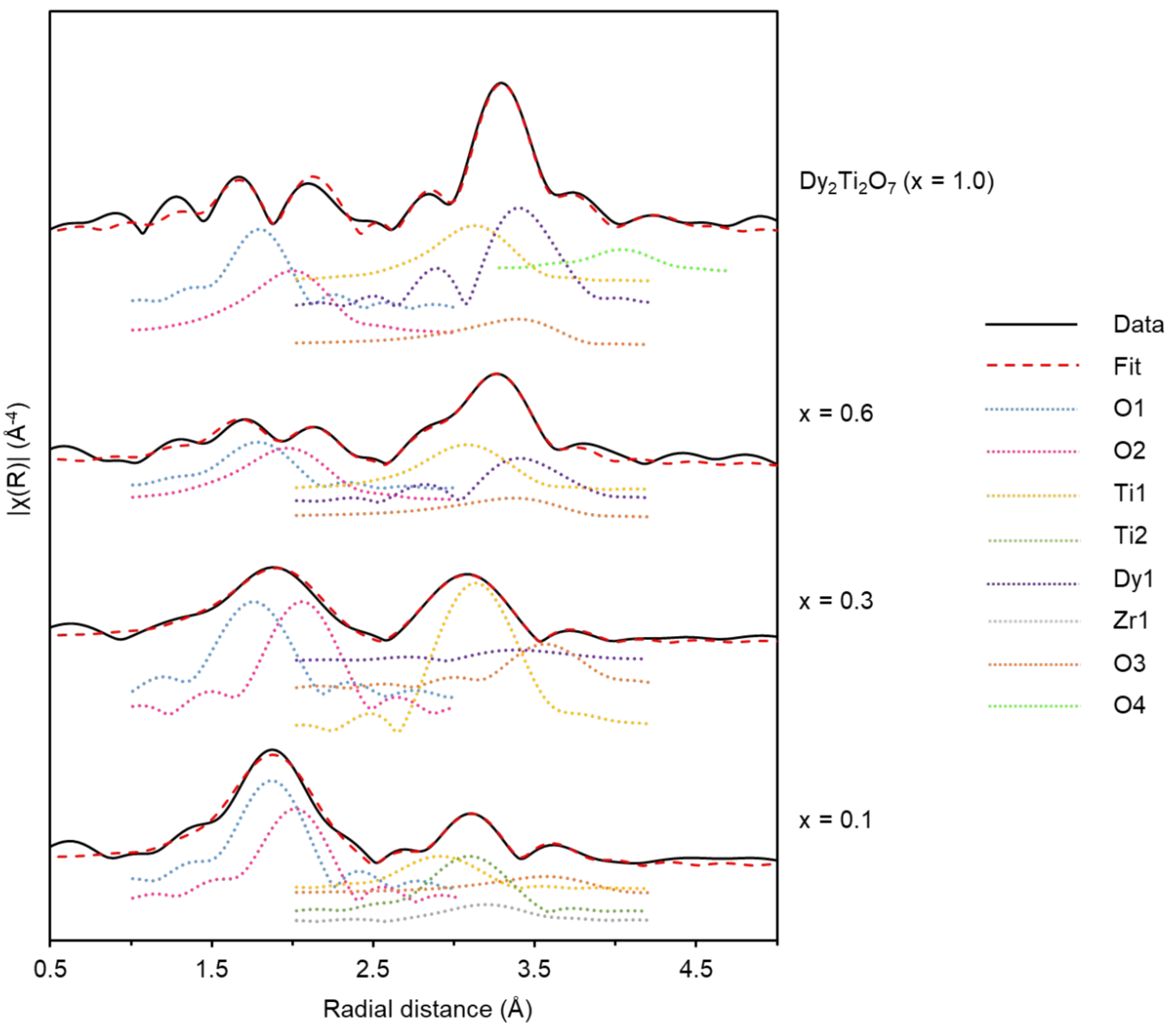

Fig. S2) Deconvoluted EXAFS spectra for all samples showing all single scatterer contributions to the fit.

Table S3) EXAFS fitting parameters for a range of fitting models for the x = 0.6 sample that include Zr and Ca backscatterers in a variety of combinations. All nomenclature used is identical to that in the main manuscript.

Sample	Parameters	Path						
		O1	O2	Ti1	Ca1	Zr1	Dy1	O3
**x = 0.6**<br>E_0 = 1.6(13)<br>R-factor = 0.0076	N	2	6	6	-	-	3.6	12
	σ^2 (10^{-3}) (Å^2)	7(2)	15(2)	15(2)	-	-	7(1)	19(7)
	R (Å)	2.21(2)	2.43(2)	3.53(1)	-	-	3.59(2)	3.93(5)
**x = 0.6 with 2.4 Zr**<br>E_0 = 0.9(28)<br>R-factor = 0.0107	N	2	6	6	-	2.4	3.6	12
	σ^2 (10^{-3}) (Å^2)	7(4)	16(4)	20(20)	-	10(19)	6(6)	12(25)
	R (Å)	2.20(4)	2.43(4)	3.56(13)	-	3.58(10)	3.59(5)	3.93(7)
**x = 0.6 with 1.2 Zr**<br>E_0 = 1.2(31)<br>R-factor = 0.0106	N	2	6	6	-	1.2	3.6	12
	σ^2 (10^{-3}) (Å^2)	7(4)	16(4)	17(16)	-	7(25)	7(7)	13(26)
	R (Å)	2.21(4)	2.43(4)	3.56(10)	-	3.56(13)	3.59(5)	3.93(8)
**x = 0.6 with 1.2 Zr + 1.2 Ca**<br>E_0 = 3.5(33)<br>R-factor = 0.0100	N	2	6	6	1.2	1.2	3.6	12
	σ^2 (10^{-3}) (Å^2)	6(4)	14(4)	1(4)	-4(7)	-5(5)	24(53)	32(31)
	R (Å)	2.22(5)	2.45(5)	3.52(3)	3.30(6)	3.48(5)	3.82(29)	4.02(20)
**x = 0.6 with 1.2 Ca**<br>E_0 = 5.4(15)<br>R-factor = 0.0108	N	2	6	6	1.2	-	3.6	12
	σ^2 (10^{-3}) (Å^2)	7(3)	15(3)	9(2)	-3(1)	-	18(11)	79(53)
	R (Å)	2.25(3)	2.48(2)	3.50(2)	3.65(2)	-	3.83(7)	4.19(41)
**x = 0.6 with 2.4 Ca**<br>E_0 = 5.7(10)<br>R-factor = 0.0080	N	2	6	6	2.4	-	3.6	12
	σ^2 (10^{-3}) (Å^2)	6(2)	15(2)	4(1)	0(1)	-	16(7)	77(68)
	R (Å)	2.25(2)	2.48(2)	3.58(1)	3.44(2)	-	3.89(4)	4.34(19)

*The amplitude reduction factor (S_0^2) for all samples was 0.95; N is the degeneracy; σ^2 is the Debye-Waller factor; R is the interatomic distance; α is the result of the F-test indicating the confidence that adding the path improves the fit (>67 % is equal to 1σ and >95 % is equal to 2σ in terms of standard deviation); §Indicates path was parameterised using O1 and Ti1 parameters; *Indicates that the parameters were linked; ¤Indicates parameters were linked. The general formula relating to 'x' is $Ca_{1-x}Zr_{1-x}Dy_{2x}Ti_2O_7$.*

As discussed in the main manuscript, fits were attempted that include contributions from Ca/Zr backscatterers but were unsuccessful. **Table S3** provides the fitting results for a range of models that aim to incorporate the contributions from Ca/Zr backscatterers in varying proportions with the best fit model (that was used in the main manuscript) given for comparison. In this case, the x = 0.6 sample is given as an example, however similar effects were observed for all samples. The addition of Ca/Zr backscatterers did not qualitatively change the fit in any significant capacity, however quantitatively all fits that included these backscatterers were either poorer fits or incorrect models.

Upon the addition of only Zr backscatterers, the fit was quantitatively invalid due to the error on the Debye-Waller factor significantly exceeding its refined value. When both Zr and Ca were added, both backscatterers refined to have negative Debye-Waller factors indicating the model is incorrect. The addition of only Ca backscatterers again refined to unrealistic and incorrect Debye-Waller factors, whilst also significantly impacting the Debye-Waller factors and interatomic distances of other shells in the fit.

Consequently, it was determined that the addition of Ca/Zr backscatterers was not feasible with this dataset.
